# Role of Nrf2 in Disease: Novel Molecular Mechanisms and Therapeutic Approaches – Pulmonary Disease/Asthma

**DOI:** 10.3389/fphys.2021.727806

**Published:** 2021-09-29

**Authors:** Camille Audousset, Toby McGovern, James G. Martin

**Affiliations:** Meakins-Christie Laboratories, McGill University, Montréal, QC, Canada

**Keywords:** Nrf2, respiratory disease, oxidative stress, inflammation, Nrf2–Keap1 pathway, asthma, molecular mechanism

## Abstract

Nuclear factor erythroid 2-related factor 2 (Nrf2) is a major transcription factor involved in redox homeostasis and in the response induced by oxidative injury. Nrf2 is present in an inactive state in the cytoplasm of cells. Its activation by internal or external stimuli, such as infections or pollution, leads to the transcription of more than 500 elements through its binding to the antioxidant response element. The lungs are particularly susceptible to factors that generate oxidative stress such as infections, allergens and hyperoxia. Nrf2 has a crucial protective role against these ROS. Oxidative stress and subsequent activation of Nrf2 have been demonstrated in many human respiratory diseases affecting the airways, including asthma and chronic obstructive pulmonary disease (COPD), or the pulmonary parenchyma such as acute respiratory distress syndrome (ARDS) and pulmonary fibrosis. Several compounds, both naturally occurring and synthetic, have been identified as Nrf2 inducers and enhance the activation of Nrf2 and expression of Nrf2-dependent genes. These inducers have proven particularly effective at reducing the severity of the oxidative stress-driven lung injury in various animal models. In humans, these compounds offer promise as potential therapeutic strategies for the management of respiratory pathologies associated with oxidative stress but there is thus far little evidence of efficacy through human trials. The purpose of this review is to summarize the involvement of Nrf2 and its inducers in ARDS, COPD, asthma and lung fibrosis in both human and in experimental models.

## Structural Features of Nrf2

The transcription factor NF-E2-related factor 2 (Nrf2) plays a central role in controlling both constitutive and inducible resistance to oxidants and electrophiles (Rushmore and Kong, 2002). Nrf2 is a master regulator of redox homeostasis, involved in the regulation of more than 500 genes, including genes that regulate oxidative stress [heme oxygenase (HO)-1, glutamate-cysteine ligase modifier subunit (GCLM), and glutamate-cysteine ligase catalytic subunit (GCLC)], inflammation [transforming growth factor (TGF)-β and Nuclear Factor Kappa (NF-*κ*)B], xenobiotic metabolism and excretion [NAD(P)H quinone oxidoreductase (NQO1), Aldo-Keto Reductase Family 1 Member C1 (AKR1C1), and multidrug resistance-associated protein (MRP)-1], apoptosis (Bcl-2 and BclxL), and autophagy (p62) (Kensler et al., 2007; Rada et al., 2012; Jaramillo and Zhang, 2013). Nrf2 is a member of the vertebrate Cap’n’Collar (CNC) transcription factor subfamily of basic leucine zipper (bZip) transcription factors. In addition to Nrf2, the CNC subfamily of transcription factors comprises nuclear factor E2-related factors 1 and 3 (Nrf1 and Nrf3), and p45 NF-E2. Nrf2 activation occurs under various conditions of stress, such as exposure to mild oxidative or electrophilic stress. Multiple classes of chemical inducers are known to elevate endogenous antioxidants by activating Nrf2 (Yamamoto et al., 2018). The most important feature of Nrf2 is its rapid mobilization and nuclear translocation that reflects the mechanisms that control its cytoplasmic free concentration.

Ubiquitin proteasome system (UPS)-mediated mechanisms direct the meticulous regulation of Nrf2. While cells are in homeostatic conditions, Nrf2 is targeted for constant degradation through the UPS. This results in low levels of free Nrf2 protein and constrains the transcription of Nrf2-dependent genes. In an unstressed state cellular degradation occurs through Kelch-like ECH-associated protein 1 (Keap1), an adaptor protein of a cullin3 (Cul3)–ring-box 1 (Rbx1) containing E3 ubiquitin ligase complex (Kobayashi et al., 2004; Zhang et al., 2004). Dimeric Keap1 is responsible for recognition of Nrf2 through two key motifs in the Neh2 domain of Nrf2 located in its N-terminus (Zhang et al., 2004). The Kelch domain of each Keap1 binds to the ‘DLG’ and ‘ETGE’ motifs, recognized as the low affinity and high-affinity-binding sites, respectively (Enomoto et al., 2001). Nrf2 is subsequently polyubiquitinylated at seven key lysine residues within the Neh2 domain, leading to Nrf2 proteasomal destruction (Dinkova-Kostova et al., 2001). Upon activation, the levels of Nrf2 rise and nuclear Nrf2 heterodimerizes with one of the small Maf proteins. These Nrf2–Maf heterodimers recognize antioxidant response elements (AREs), 11- (or 16) bp enhancer sequences in the regulatory region of Nrf2 target genes, thereby allowing the recruitment of key factors for transcription (Harder et al., 2015). Typically, genes that contain the ARE are redox-balancing factors, detoxifying enzymes, transporters, stress-response proteins and metabolic enzymes (Kensler et al., 2007; Malhotra et al., 2010). Activation of Nrf2 occurs primarily through increased stability of the Nrf2 protein resulting in enhanced availability for binding to the ARE. Subsequently, this allows for increased transcription of the antioxidant genes without the requirement for the synthesis of Nrf2 *de novo*.

### Keap1-Dependent Regulation of Nrf2 Activity

The most studied and widely accepted mechanism of Nrf2 regulation is through Keap1–Cul3–Rbx1 E3 ubiquitin ligase. Many reactive oxygen species (ROS) are thiol-reactive compounds that inhibit Keap1 activity. Exposure of thiol-reactive compounds induces a conformational change in Keap1, impeding the ubiquitinylation of Nrf2 by Cul3. Upon introduction of electrophiles or ROS, the Nrf2-mediated cytoprotective response is activated. Although several mechanisms have been proposed, explicit molecular mechanisms detailing how Nrf2 escapes the Keap1 gate are not fully understood. One such mechanism involves the modification of a cysteine in Keap1 resulting in Nrf2 dissociation from Keap1 (Baird and Dinkova-Kostova, 2011). A more widely accepted model proposes a Keap1 hinge and latch dissociation mechanism in which Nrf2 binds to the Keap1 homodimer through a high-affinity ETGE motif as the “hinge” and a low-affinity DLG motif as the “latch.” The modification of cysteine in Keap1 results in a conformational change but does not trigger the dissociation of Nrf2. Ubiquitin binding to Nrf2 may therefore disrupt the weak latch binding site (Tong et al., 2006). Activation of Nrf2 by Keap1 ubiquitinylation has also been proposed and involves the modification of a cysteine in Keap1 which allows the ubiquitin conjugation from Nrf2 to itself (Dinkova-Kostova et al., 2001). The likely critical cysteine residues in Keap1 that play a role have been identified. Specifically, cysteine residues in Keap1, especially Cys^151^, have been shown to act as sensors and become covalently modified by electrophilic species or ROS (Zhang and Hannink, 2003). Other studies have shown a role for Cys-273 and Cys-288 in Keap1 which appear to contribute to the structural integrity and activity of Keap1 required for maintaining ubiquitin ligase activity (Kobayashi et al., 2006). Such modifications induce a conformational change in Keap1, probably by disrupting the low-affinity interaction between the Kelch domain and the DLG-motif, which leads to impaired ubiquitinylation of Nrf2, blocking UPS-mediated degradation and thus increasing Nrf2 protein levels (Baird et al., 2013). Newly synthesized Nrf2 accumulates as a result of the altered stoichiometry and cytosolic Nrf2 is then free to translocate into the nucleus and transcriptionally activate its target genes. Once homeostasis is restored in the cell, karyopherin alpha 6 (importin alpha 7) (KPNA6) translocates Keap1 to the nucleus. Nuclear Keap1 facilitates nuclear export of Nrf2, and the cycle of Keap1-mediated ubiquitinylation and degradation in the cytosol resumes. These events effectively “turn off” the transcription of the Nrf2 target genes, and the low basal levels of free Nrf2 are re-established (Sun et al., 2007, 2011).

### Keap1-Independent Regulation of Nrf2 Activity

Keap1-independent mechanisms that can control Nrf2 activity have been identified. In 2004, a redox-insensitive degron within the Neh6 domain of Nrf2 was reported (McMahon et al., 2004) followed by the discovery that the Neh6 domain of mouse Nrf2 contains a group of serine residues that can be phosphorylated by the serine/threonine kinase glycogen synthase kinase 3 (GSK-3). This phosphorylation event in the Neh6 domain creates a phosphorylated destruction motif (phosphodegron), which can then be recognized by the β-TrCP–Skp1–Cul1–Rbx1 E3 ubiquitin ligase complex (Rada et al., 2011). Additionally, Nrf2 can be regulated by other proteins through disruption of the Nrf2–Keap1 interaction. Hast et al. (2013) identified a stabilizing mechanism for Nrf2 which included numerous proteins with motifs identical (or similar) to the ETGE motif of Nrf2. These proteins were shown to compete with Nrf2 for Keap1 binding, resulting in the stabilizing effect (Hast et al., 2013). Protein kinases have also been shown to play an essential role in Keap1-independent Nrf2 activation where phosphorylation at a specific amino acid residue of Nrf2 can increase its stability and transactivation activity (Nguyen et al., 2003). Identified protein kinase pathways associated with Keap1-independent activation of Nrf2 include phosphatidylinositol 3-kinase (PI3K), MAPKs, PKC, and glycogen synthase kinase-3 (GSK-3) (Liu et al., 2019). PI3K, PKC, c-Jun, N-terminal kinase (JNK) and extracellular signal-regulated protein kinase (ERK) have all been shown to positively regulate Nrf2 (Tong et al., 2006), whereas p38 MAPK regulates the Nrf2 pathway both positively and negatively (Yu et al., 1999, 2000; Kobayashi et al., 2006; Rojo et al., 2008). Other positive regulators of Nrf2 include (E/S)TGE containing proteins such as dipeptidyl peptidase 3 (DPP3) and partner and localizer of BRCA2 (PALB2), although perhaps the most recognized positive regulator of Nrf2 involves p62, a protein containing the STGE motif (Harder et al., 2015).

### Transcriptional Regulation of Nrf2

Nrf2 can also be transcriptionally regulated through oncogenes; Nrf2 can be up-regulated by oncogenic activation of K-Ras^*G12D*^, B-Raf^*V619E*^, and Myc^*ERT2*^ (DeNicola et al., 2011). The first demonstration of Nrf2 modulation at the transcriptional level was shown through activation of Nrf2 by oncogenic K-rat sarcoma (Ras) and facilitated through a 12-*O*-Tetradecanoylphorbol-13-acetate (TPA)-responsive element (TRE) in the regulatory region of *Nrf2* (Tao et al., 2014). Although the precise mechanisms by which B-Raf^*V619E*^ and Myc^*ERT2*^ up-regulate the transcription of Nrf2 are currently unknown, these studies suggest a possible therapeutic benefit of Nrf2 inhibitors, such as brusatol, in this context to facilitate chemotherapy (Cai et al., 2019).

## Nrf2 and Disease

### Acute Respiratory Distress Syndrome and Nrf2

Acute respiratory distress syndrome (ARDS) describes a severe clinical syndrome defined by acute respiratory failure with substantial morbidity and mortality. Although the etiology has proven complex with a high degree of variability, severe pneumonia, sepsis, viral infections, major traumas, transfusions, and inhalation of noxious substances are some of the major causes. Clinical symptoms include dyspnea, rapid breathing, hypoxemia, and bilateral opacities on a chest radiograph (Ferguson et al., 2012; Fan et al., 2018). Despite the various etiologies leading to ARDS, many of the hallmark signs and symptoms are shared. In the acute phase of ARDS (days 1–6), rapid onset of widespread inflammation in the lungs and the disruption of the epithelial-vascular barrier caused by the loss of epithelial and endothelial integrity results in interstitial and alveolar edema (Johnson and Matthay, 2010). Pulmonary inflammation is characterized by large numbers of neutrophils and macrophages and epithelial cell damage is often observed as a denudation of airways and alveolar spaces (Potey et al., 2019). The subacute phase (days 7–14) typically show reabsorption of edema, and evidence of repair in the form of cell proliferation, particularly of alveolar type II cells. Neutrophilia persists and infiltration of fibroblasts may be observed at this point as well as evidence of collagen deposition (Matthay and Zemans, 2011). In the chronic phase (day 14+) neutrophil numbers wane and are replaced by an increase in mononuclear cells, alveolar macrophages and increasing fibrosis. The consequence of these pathologies results in immediate, severe airway damage and dysfunction often with long term lung function impairment (Matthay and Zemans, 2011).

The mechanism driving the pathogenesis of ARDS remains elusive and therefore specific, effective therapeutics are lacking. However, several studies have shown there to be two recurring, indispensible and interactive factors, namely, lung inflammation and oxidative stress. It is well established that oxidative injury to the lung can be largely mediated by Rocksen et al. (2003). Biologically important ROS include superoxide anion radical (O_2_^–^), hydrogen peroxide (H_2_O_2_), hydroxyl radical (OH^–^), and hypochlorous acid (HOCl) (Chow et al., 2003). Additionally, reactive nitrogen species, including peroxynitrite (ONOO–), a derivative of nitric oxide, have also been shown to contribute to ARDS pathogenesis (Haddad et al., 1994; Ischiropoulos, 1998). The generation and presence of ROS and nitrogen species leads to tissue damage, cell injury and cell death by several mechanisms. Strand breaks and point mutations can directly damage DNA leading to an increase in lipid peroxidation, resulting in the formation of vasoactive and proinflammatory molecules such as thromboxane (Gentile et al., 2017). Oxidation of proteins, particularly at sulfhydryl groups, can alter their activity (Fialkow et al., 1993). Presence of oxidized proteins induces the release of proteases, inactivation of antioxidants and reduced production of antiprotease enzymes (Griffiths, 2000). Finally, oxidation can enhance the activity of transcription factors, such as activator protein-1 (Chaum et al., 2009) and NF-κB (Lingappan, 2018), leading to enhanced expression of proinflammatory genes (Hong et al., 2013).

**FIGURE 1:**
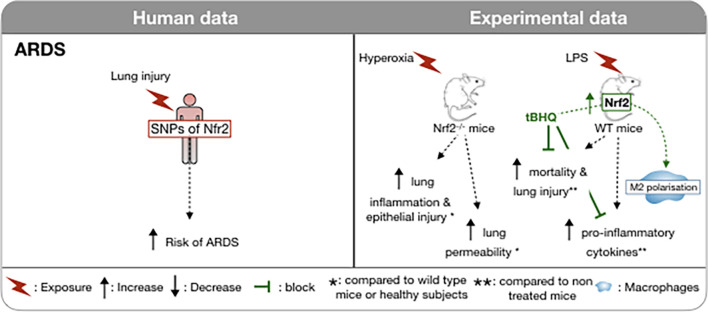
Involvement of Nrf2 in ARDS in humans and *in vivo* models. In humans, the risk of developing an ARDS during severe trauma is associated with the possession of a functional promoter that has affected the binding affinity to a promoter site upstream of the Nrf2 gene. Induction of hyperoxia-induced ARDS in NRf2-deficient mice is associated with increased lung inflammation and capillary permeability. Conversely, in a LPS induced ARDS model, induction of Nrf2 is associated with a decrease in mortality and inflammation, notably via the M2 polarization of macrophages. **↑,** Increase; **↓,** Decrease; 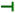 block; *, compared to wild type mice or healthy subjects; **, compared to un- treated mice; 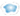, macrophages; WT, wild type.

Cells synthesize a number of endogenous Nrf2-dependent antioxidants including superoxide dismutase, catalase, and glutathione peroxidase designed to neutralize ROS and mitigate damage during ARDS (Fink, 2002). Several studies in both animals and humans have demonstrated the importance of Nrf2 activity in the context of ARDS. In a hyperoxia-induced murine model of ARDS, Nrf2 has been identified as a susceptibility gene, as single nucleotide polymorphisms (SNPs) have been found in hyperoxia-susceptible C57BL/6J and hyperoxia-resistant C3H/HeJ mice (Cho, 2013). Nrf2-deficient mice have demonstrated an increased likelihood of developing ARDS, and have enhanced lung hyperpermeability, epithelial injury, and inflammation following exposure to hyperoxia or treatment with butylated hydroxytoluene (Chan and Kan, 1999; Cho et al., 2002). Not surprisingly, basal and induced expression levels of Nrf2-dependent genes were reduced in Nrf2^–/–^ mice suggesting that Nrf2 transcriptional activity is key in the response to hyperoxia-induced acute lung injury (Rojo de la Vega et al., 2016). In humans, over 500 single-nucleotide polymorphisms (SNPs) of Nrf2 have been identified to date. Importantly, the risk of developing ARDS after severe trauma was shown to be enhanced in European and African American individuals possessing a functional Nrf2 SNP (Figure 1) that affected the binding affinity at a promoter site upstream of the Nrf2 gene (Marzec et al., 2007). This study also suggested that Nrf2 autoregulated its expression via binding to this site.

Recently, studies have focused on exploiting Nrf2 activity using various Nrf2 activators in an effort to protect against the effects of ARDS. The Nrf2 activator, oleanane triterpenoid CDDO-imidazole (CDDO-Im) is reported to activate Nrf2/ARE signaling by interfering with the interaction between Keap1 and Nrf2 in the cytosol (Sussan et al., 2009). CDDO-Im inhibits pulmonary hemorrhage, proteinaceous edema, and inflammatory cell infiltration in hyperoxia-induced ARDS in mice (Reddy et al., 2009). Another Nrf2 activator, tert-butylhydroquinone (tBHQ), was shown to reduce lipopolysaccharide (LPS)-induced mortality and lung injury in mice and to down-regulate pro-inflammatory mediators and up-regulate anti-inflammatory mediators (Figure 1; Wei et al., 2018). Interestingly, it appears that tBHQ confers its protection primarily by promoting the polarization of M2 macrophages by inhibiting the p65 nuclear factor-κB pathway and activating peroxisome proliferator-activated receptor-γ, while suppressing the polarization of M1 macrophages. Resveratrol is a dietary polyphenol found in wine and red-skinned fruit with many anti-inflammatory properties and it also activates Nrf2. Similarly to tBHQ, resveratrol has been shown to suppress the M1 macrophage population as well as several pro-inflammatory cytokines and chemokines [tunour necrosis factor (TNF)-α, interleukin (IL)-1β, IL-6, chemokine (C-C motif) ligand 2 (CCL2)/monocyte chemoattractant protein (MCP)-1, CCL4/macrophage inflammatory protein-1β (MIP1β), CCL5/Regulated on activation, normal T cell expressed and secreted (RANTES) and CXCL10/Interferon gamma-induced protein 10 (IP-10)] following LPS-induced acute lung injury in mice (Hu et al., 2019). Perhaps the most well-studied Nrf2 activator is sulforaphane (SFN), a naturally occurring sulfur-rich isothiocyanate found in cruciferous vegetables. While the precise mechanisms by which SFN acts as an Nrf2 activator are not fully understood, the chemical properties of SFN suggest direct interaction with Keap1. Cysteine residues with low pKa values are particularly reactive with isothiocyanates. These cysteines are thiolate anions at physiological pH, and therefore primed for efficient nucleophilic attack on electrophilic substrates. Upon entry into the cell, SFN interacts with Keap1, which contains a number of cysteine residues. These residues are sensors for oxidants and electrophiles, including isothiocyanates (Dinkova-Kostova et al., 2017). SFN triggers a conformational change in Keap1, freeing Nrf2 and allowing it to translocate to the nucleus, where it binds to the ARE and initiates the subsequent transcription of antioxidants begins. In addition to activating Nrf2 directly through interaction with Keap1, SFN may also protect against ARDS indirectly. In a mouse model of ARDS induced by LPS, SFN was shown to inhibit the increase of NF-κB, prevent increases in several proinflammatory mediators and enzymes cyclooxygenase-2 (COX-2) and matrix metalloproteinase-9 (MMP-9) as well as activating the Nrf2 pathway (Qi et al., 2016). As with most of the pathologies that we discuss further in this review, there is a lack of favorable evidence through clinical trials in human ARDS and in some instances outcomes suggest possible harmful effects of treatments (Bo et al., 2021).

### Nrf2 and Chronic Obstructive Pulmonary Disease

Chronic obstructive pulmonary disease (COPD) contributes significantly to the global burden of disease as one of the main causes of preventable morbidity and mortality worldwide (Lozano et al., 2012). COPD is a slowly progressive disease characterized by persistent airflow obstruction, dyspnea, wheeze, productive cough, and increased susceptibility to respiratory infection. The progressive decline of pulmonary function, mostly frequently assessed from the forced expiratory volume in one second (FEV_1_), is a characteristic of COPD and may lead to chronic respiratory failure and death. Decline in lung function has been linked to polymorphisms of Nrf2-related genes and susceptibility to cigarette smoke (CS)-induced decline in lung function (reviewed in Okeleji et al., 2021). Acute exacerbations of respiratory symptoms are also a typical and important feature of COPD. They can be triggered by infection, pollution or other factors (Singh et al., 2019). Several factors are proposed to contribute to the development and pathogenesis of COPD but interactions between host genetic susceptibility and exposure to particulates are the most widely accepted mechanisms. Genetic risk factors associated with an increased risk of COPD are alpha antitrypsin deficiency in smokers, represented by the homozygous PiZZ and heterozygous PiMZ genotype (Molloy et al., 2014) and an elastin mutation (Kelleher et al., 2005). Other associations have been demonstrated with GWAS studies but consistently replicated (Barnes et al., 2015). The postulated pathogenesis of COPD includes protease-anti-protease imbalance, recruitment of inflammatory cells, peri-bronchiolar fibrosis and oxidative stress.

**FIGURE 2:**
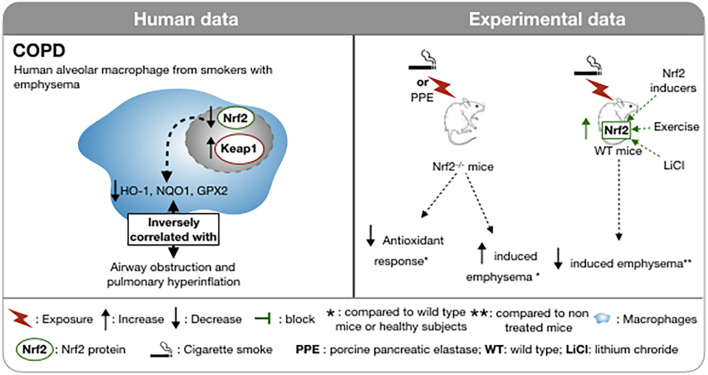
Involvement of Nrf2 in COPD in human and in experimental models. In smokers with emphysema, the nuclear localization of Nrf2 is decreased in macrophages as well as the anti-oxidant response. This altered antioxidant response is inversely correlated with airflow obstruction. In Nrf2 deficient mice, CSE or pancreatic elastase intra-tracheal instillations evoke a decreased antioxidant response and an increase emphysema. In Nrf2-deficient mice, CSE or intra-tracheal instillation of pancreatic elastase results in a decreased antioxidant response and increased emphysema compared to wild type mice. Upregulation of Nrf2 by physical activity, LiCl or Nrf2 inducers reduces the induction of cigarette smoke-induced emphysema. **↑,** Increase; **↓,** Decrease; 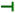, block; *, compared to wild type mice or healthy subject; **, compared to non treated mice; 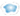, macrophages; 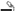, Cigarette smoke; WT, wild type; PPE, porcine pancreatic elastase; WT, wild type; LiCl, lithium chloride.

Cigarette smoke exposure, in particular, has been identified as the primary and most preventable cause of COPD (Kohansal et al., 2009). A single puff of CS contains more than 1 × 10^15^ oxidant molecules (Pryor and Stone, 1993). In the rat, chronic exposure to CS induced an up-regulation of the anti-oxidant genes controlled by Nrf2 and development of a COPD-like phenotype (Gebel et al., 2004). Surprisingly, in smokers with emphysema, the nuclear localization of Nrf2 and the expression of HO-1 are decreased in the lung and, more specifically in the macrophage population compared to healthy subjects and to smokers without emphysema (Figure 2; Goven et al., 2008). This decrease in the nuclear localization of Nrf2 in macrophages is associated with an increase in the expression of both Keap1 and Bach1, a repressor of HO-1 expression (Alam and Cook, 2007), in smokers with emphysema. HO-1 mRNA level is significantly negatively correlated with airways obstruction and lung hyperinflation (Goven et al., 2008). CSE treatment of murine macrophages inhibits LPS-induced expression of the scavenger receptor MARCO (Macrophage Receptor with Collagenous Structure) but concomitantly promotes the degradation of Keap1. The resultant increase in nuclear translocation of Nrf2 is offset by interference with Nrf2 acetylation that impairs DNA binding of Nrf2. Proteasome inhibitors prevent the suppression of MARCO expression. Thus, the effects of CSE are complex and overall interfere with a favorable host response to oxidative stress and immunity (Lee et al., 2021).

On the genetic level, the study of the structure of the Nrf2 gene on whole blood samples revealed three single nucleotide polymorphisms and one triplet repeat polymorphism in the gene promoter, although without a strong correlation with COPD in this study (Yamamoto et al., 2004). Such a link with COPD has been made by other investigators that observed that the haplotype of the Nrf2 gene promoter affects its activity and may also be associated with more severe COPD and risk of respiratory failure (Hua et al., 2010). In addition, several studies suggest a modification of the activation of the Nrf2 pathway depending on the cell type and smoking status in COPD patients. Alveolar macrophages of patients with COPD exhibit less nuclear and cytosolic Nrf2 protein (Goven et al., 2008) and less Nrf2 mRNA compared to healthy subjects (Suzuki et al., 2008). In peripheral blood mononuclear cells of mild-moderate COPD patients, regardless of smoking status, the expression level of Nrf2 and Nrf2-related genes is increased (Fratta Pasini et al., 2016; Fratta Pasini et al., 2020). However, interestingly in COPD patients, after an average follow-up of 49.7 ± 6.9 months, a decline in Nrf2 expression in PBMC was observed and correlated with a significant decrease in respiratory function, as measured by FEV_1_ (Fratta Pasini et al., 2020). In addition, the smoking status (former or current smoker) in COPD patients may affect the level of transcription of Nrf2 target genes (NQO1, HO1, AKR1C1, AKR1C3) in the bronchial epithelium, in nasal epithelial cells (AKR1B10) and in peripheral blood mononuclear cells (HO-1) whereas none is altered in alveolar macrophages (Sidhaye et al., 2019).

The above findings are supported by numerous *in vitro* and *ex vivo* studies. Nrf2–/– mice exposed to CS develop earlier onset emphysema (Rangasamy et al., 2004; Iizuka et al., 2005), increased markers of oxidative stress, as assessed by 8-oxo-dG positive cells and more severe inflammation revealed by bronchoalveolar lavage (Rangasamy et al., 2004). In addition, the up-regulation of antioxidant enzymes, including HO-1, glutathione- S-transferase-α1 (GST-α1), G6PD, glutathione reductase (GSR) and peroxiredoxin I (PrxI) induced by CS in wild type mice was not found in Nrf2–/– mice (Figure 2; Rangasamy et al., 2004; Iizuka et al., 2005). In agreement with these data, activation of Nrf2 by a selective inhibition of Keap1 in club cells in mice, leads to an increased amount of glutathione (GSH) and NQO1 in the lung and an attenuation of pulmonary inflammation caused by CS (Blake et al., 2010). *In vitro*, inhibition of Keap1 by siRNA in human bronchial epithelial cells exposed to hydrogen peroxide mitigates the oxidative stress assessed by DCFDA fluorescence (Blake et al., 2010). As CS exposure is not the unique pathologic mechanism for COPD, the involvement of Nrf2 in the pathogenesis of other models of emphysema has been tested. Protease/anti-protease imbalance and oxidative stress may also participate in the formation of emphysema (Barnes et al., 2003) and has led to the development of protease-induced emphysema murine models (Hayes et al., 1975). Intra-tracheal injection of porcine pancreatic elastase (PPE) in mice induces a robust pulmonary inflammation and alveolar destruction (Figure 1). This exposure triggers a strong antioxidant response by increasing NQO1, GST-Yc, HO-1, and PrxI. In Nrf2^–/–^ mice, the PPE-induced emphysema is more severe and, as expected, the antioxidant response is reduced. Transplantation of WT bone marrow cells into Nrf2^–/–^ mice 14 days prior to treatment by PPE delays the initial pulmonary inflammation and the appearance of emphysema, attributed to the presence of Nrf2 expressing macrophages in lung (Ishii et al., 2005). Furthermore, in two mouse models of COPD induced either by cigarettes or by orotracheal instillation of PPE (Wright et al., 2008), the expression of the WNT/β-catenin signaling pathway is significantly decreased. In these models, preventive or curative administration of an agonist of the WNT/β-catenin pathways, lithium chloride (LiCl), improves lung function and limits airspace enlargement (Kneidinger et al., 2011). Recently, the protective effect of WNT/β-catenin activation by LiCl has been shown to be abrogated in an Nrf2^–/–^ mouse model of elastase-induced emphysema. Nrf2 is a downstream signal in the protective effect of Wnt3a/β-catenin and AMP-activated protein kinase (AMPK) in the attenuated lung inflammatory response (Cui et al., 2018).

The above-mentioned data suggest that Nrf2 is involved in the antioxidant response to several underlying pathogenic mechanisms of COPD. However, COPD is a disease with systemic features and the involvement of Nrf2 is not limited to the regulation of the antioxidant response. For instance, CS exposure triggers airway inflammation and can result in cilia shortening, impaired mucociliary clearance and epithelial cell dysfunction (Leopold et al., 2009). Ciliary shortening is mediated by histone deacetylases 6 (HDAC6) through an autophagy-dependent mechanism (Lam et al., 2013). However, it has been shown that Nrf2 inhibition may also increase autophagy (Rao et al., 2010). In Nrf2^–/–^ mice, the level of HDAC6 was increased, suggesting that Nrf2 could counteract the increased expression of HDAC6 by oxidative stress and proteolytic stress (Lam et al., 2013).

Skeletal muscle dysfunction is among the systemic effects of COPD (Jaitovich and Barreiro, 2018). Recent work has shown that the level of irisin, a skeletal muscle cell-derived hormone, is correlated with the severity of emphysema in COPD patients and that it inhibits apoptosis induced by CS on human A549 alveolar cells (Sugiyama et al., 2017). Physical activity in mice exposed to CSE increases the levels of irisin that is associated with an increased pulmonary expression of Nrf2 and HO-1 and less emphysema (Kubo et al., 2019).

Nrf2 is involved in acute infectious exacerbations and in corticosteroid resistance, two major characteristics of COPD. Respiratory viruses, such as influenza, are responsible for severe infectious exacerbations in COPD patients. Nrf2^–/–^ mice exposed to CS and subsequently infected with influenza have a higher rate of mortality, an enhanced peribronchial inflammation, a higher oxidant burden and NF-κB-mediated inflammatory gene expression (Yageta et al., 2011). Interestingly, knockdown of Nrf2 in human nasal epithelial cells (hNEC) is associated with a significantly increased influenza virus entry and replication in the infected cells whereas the Nrf2 activators, SFN and epigallocatechin gallate treatment of native hNEC cell significantly reduce the viral entry and replication (Kesic et al., 2011). Therefore, Nrf2 activation is a plausible therapeutic strategy to reduce the sensitivity of COPD patients to viral infections.

Bacteria, especially *non-typeable Haemophilus influenzae* (NTHI), can cause acute exacerbations of COPD. In mice with NTHI infection, prior exposure to CS for two weeks enhances BAL macrophage and neutrophil recruitment, greater oxidative stress and pulmonary inflammation compared to infected but air-exposed mice (Jaitovich and Barreiro, 2018). Andrographolide is a labdane diterpene lactone extracted from the leaf of *Andragraphis paniculata*, a medicinal herbal used in Asian countries, which enhances Nrf2 translocation to the nucleus (Yu et al., 2010). Administered after the NTHI infection it attenuates the lung inflammation in treated mice and it up-regulates the translocation of Nrf2 into the nucleus, enhances HO-1, glutathione reductase (GSR), GCLM, glutathione peroxidase-2 (GPx-2), and NQO1 gene expression in the lungs (Table 1; Tan et al., 2016). The evidence, in particular from murine models, supports the potential for a role for Nrf2 in the exacerbations of COPD.

**TABLE 1:** Effect of Nrf2 modulation on COPD characteristics.

**Bioactive compound**	**Treatment administration**	**Species**	**Model**	**Outcomes**	↑ **antioxidant response**	**References**
SFN	10 μM for 16 h 0.5 mg/day per mouse during 3 days, nb	Human macrophage Nrf2 ^+/+^ and Nrf2^–/–^ mice	Macrophage from COPD patients Exposure to CS (for 1 week or 6 months), challenge with PA 24 h after SFN treatment	In macrophages from COPD subjects: - ↑ bacterial clearance of PA and NTHI through an enhancement of phagocytosis - ↑ increase the expression of the scavenger receptor MARCO In mouse model: - ↓ the bacterial burden and ↑ the phagocytosis of macrophage in Nrf2^+/+^ but not in Nrf2^–/–^ mice	Nrf2^*b*^, NQO1^*a*^, GPX2^*a*,^	Harvey et al., 2011
	Pre-treatment with 0.5 μM for 12 h	Rat epithelial type II cells (RLE-6TN)	Exposure to different concentrations of CSE (1–20%) for 24 h	- ↓ cell death by apoptosis and ↓ G1 phase cells cycle arrest - ↓ CS induced ROS production	Nrf2^*a,b*^	Jiao et al., 2017
	25 or 125 μmoles/day, orally for 4 weeks	COPD Subjects	Parallel, placebo control, phase 2 trial	- No significant difference between groups in Nrf2 expression in alveolar macrophages and bronchial epithelial cells - Clinically well tolerated	None	Wise et al., 2016
CDDO-Im	60 or 90 mg/kg diet throughout CS exposure	Nrf2^+/+^ and Nrf2^–/–^ mice	Exposure to CS for 5 days/week for 6 month	- ↓ alveolar destruction (MLI) in Nrf2 ^+/+^ but not in Nrf2^–/–^ mice - ↓ oxidative stress (OHdG) through with an increase glutathione production in Nrf2^+/+^ mice only - ↓ CS induced apoptosis (TUNEL-positive cells) in both strains of mice	HO-1^*a*^, NQO1^*a*^, Gclc^*a*^, Gclm^*a*^, Srx1^*a*^, G6PD^*a*^	Sussan et al., 2009
Resveratrol	10 μM during 24 h	A549 and SAE cells	1, 2.5, and 5% CSE exposure for 24 h	- ↓ CSE induced ROS production - ↑ GCLC and restored GSH level - ↑ nuclear translocation of Nrf2 ↓ CSE induced post transcriptional modification of Nrf2/Keap1 (tyrosine nitration)	Nrf2^*bd*^; GCL^*a*^	Kode et al., 2008
Alantolactone	1, 5, 10 μM pre-treatment for 2 h before CSE exposure	Beas-2B and NHBE cells	5% CSE exposure for 24h	- ↓ IL-1 β, TNF-α, IL-6 and IFN-γ CSE induced production - ↓ caspase 3 activity and apoptosis - ↓ ROS and MDA content - ↓ CS induced phosphorylation of p65 of the NF-κB pathway	Nrf2^*b*^; HO-1^*b*^	Dang et al., 2020
Andrographolide	0.1, 0.5, and 1 mg⋅kg–1, 2 h	BALB/c mice	Exposure to CS for 1 h during 5 days, 2 h after treatment	- ↓neutrophil and total cell recruitment - ↓ lung protein level of IL-1β, MCP-1, KC and IP-10 - ↓ cytokine production of MIP-2α, MMP12, TIMP-1, GMCSF, TNFα - ↓ oxidative stress (8-Isoprostane, 8-OHdG and 3-NT levels)	Nrf2^bd^, GCLM^a^, GCLC^a^, Gpx-2^a^, HO-1^a^, CAT^c^, SOD^c^, GPx^c^, GR^c^	Guan et al., 2013
	5 mg/kg and 10 mg/kg, ip	BALB/c mice	Exposure to CS twice/days, 5 days/week during 2 week Treatment 2 h prior NTHi challenge	- ↓ total cell count, neutrophils and macrophage in the BAL - ↓ lung inflammation induced by NTHi infection in the context of CS exposure - ↓ TNF-α, IL1-β - Change the protease anti-protease balance by ↓ MMP9 and MMP8	Nrf2^b^, GCLM^a^, Gpx-2^a^, HO-^a^, NQO1^a^, GR^a^	Tan et al., 2016
Triterpene Acids	50 mg/kg or 100 mg/kg, ig from the 7 weeks CS exposure	C57BL/6J mice	Exposure to CS, 4 times/day for 5 days/week during 12 weeks	- ↓ lung weight assessed by the lung index* and the lung alteration assessed by MLI and DI - ↓ IL-1β_, IL-2, IL-6, and TNF-α - ↓ CS induced inflammation by ↓ p-NFκB - ↑ phosphorylation of AMPc	Nrf2^*b*^	Jian et al., 2020

nb, nebulization; ip, intraperitoneally; iv, intravenously; it, intratracheal; ig, intragastric; w, week; m, month; CS, cigarette smoke; CSE, cigarette smoke extract. A549, human type II alveolar epithelial cell line; SAEC cells, Human primary small airway epithelial cells; NHBE, normal human bronchial epithelial cells; TIMP-1, tissue inhibitor of metalloproteinases-1; MIP-2, macrophage inflammatory protein 2- α; 3-NT, 3-nitrotyrosine; GSH, glutathione; GSL, Glutamate-cysteine ligase; GCLC, glutamate-cysteine ligase catalytic; GCLM, glutamate-cysteine ligase modifier; GPx, glutathione peroxidase; GR, glutathione reductase; MARCO, Macrophage Receptor With Collagenous Structure^*a*^ mRNA expression; ^*b*^ protein expression; ^*c*^ enzymatic activity; ^*d*^nuclear localization, *Lung index (%) = lung weight (mg)/body weight (g) × 100; MLI, mean linear intercept; DI, destructive index.

Airway inflammation in COPD is characterized by corticosteroid insensitivity. This characteristic may be linked to the phosphorylation and the degradation of HDAC2 by the activation of the PI3K/Akt pathway (To et al., 2010). In Nrf2^–/–^ mice exposed to CS smoke and LPS, the loss of Nrf2 is associated with a steroid-insensitive pulmonary inflammation. Nrf2–/– mice exhibit a decreased HDAC2 activity and level in the lung at baseline and after CS and LPS challenge (Adenuga et al., 2010). In COPD peripheral blood mononuclear cells and in CS-exposed mouse lung, the phosphorylation of Akt and p70s6K are increased and the HDAC2 level and its activity are reduced by phosphorylation. Andrographolide prevents the phosphorylation of Akt, p70S6K and HDAC2 induced by CS exposure. This inhibition leads to an enhancement of HDAC2 activity and restores the steroid sensitivity of the lung. Andrographolide increases the nuclear localization of Nrf2 and the up-regulation of antioxidant genes, HO-1 (decycling) 1 (HMOX1) and NQO1 (Liao et al., 2020). The above results suggest that the Nrf2-HDAC2 axis is one of the mechanisms involved in the steroid resistance of COPD.

Targeting the interactions between Nrf2 and Keap1 may be a potential therapeutic approach in COPD. Currently, the Nrf2 inducers target the Nrf2-Keap1 interaction (Magesh et al., 2012). SFN administration to the alveolar macrophages of COPD patients improves their phagocytic capacity for bacteria such as *Pseudomonas aeruginosa* and NTHI. Nrf2 siRNA treatment of macrophages of COPD patients and of a Nrf2–/– murine model of COPD has confirmed improved bacterial clearance secondary to an Nrf2-dependent up-regulation of MARCO (Harvey et al., 2011). Furthermore, SFN decreased the oxidative stress induced by CS extract in rat epithelial cells through the Nrf2 pathway (Jiao et al., 2017). Unfortunately, daily oral administration of SFN for a month in COPD patients failed to increase the expression of Nrf2 in alveolar and bronchial epithelial cells in a randomized, double blind, placebo-controlled phase 3 trial (Wise et al., 2016). In cells harvested from COPD patients and in related *in vivo* models, the pharmacological up-regulation of Nrf2 enhances the anti-infectious response against the most frequent bacterial pathogens but to date these effects have not been confirmed in intact humans.

In rats, resveratrol inhibited LPS induced pulmonary inflammation (Table 1; Birrell et al., 2005). Human alveolar epithelial cells (A549) exposed to CS and treated with resveratrol exhibited a significant decrease in ROS production and restored GSH depletion by an Nrf2-dependent induction of glutamate-cysteine ligase (GCL) (Kode et al., 2008). The ability of resveratrol to induce Nrf2 activation has recently been confirmed but its therapeutic use is compromised by its low bioavailability and its low efficacy (Wang et al., 2019). Topical administration of resveratrol by intra-tracheal instillation has been performed once a month and reduces accelerated lung aging in telomerase-deficient mice (Navarro et al., 2017), suggesting an alternative route of administration might achieve superior results. Recently a new Nrf2 activator, the *trans*-4, 4′dihydroxystilbene (DHS), derived from resveratrol has been administered intra-peritoneally and it decreases the markers of oxidation (malondialdehyde, 8-oxo-dG), alleviates fibrosis and leukocyte infiltration by activating Nrf2 response in CS exposed mice (Wang et al., 2019).

Natural triterpenes and the synthetic triterpenoid CDDO-Im are inducers of Nrf2 (Table 1). Thus, mice, chronically exposed to CS and fed with CDDO-Im, have significantly reduced pulmonary oxidative stress assessed by 8-OHdG positive cells, alveolar destruction and pulmonary hypertension. This protective effect was abolished in Nrf2–/– mice (Sussan et al., 2009) suggesting a potential therapeutic effect of the Nrf2 inducer. Despite this promising *in vivo* result, the molecule cannot be used in humans due to its toxicity. Indeed, a phase 3 clinical trial in diabetic nephropathy was prematurely stopped for increased mortality and adverse effects (Rossing, 2013). However, new natural triterpenes like triterpene acid derived from oak leaf have been discovered and reduce CS-induced inflammation in mice (Jian et al., 2020).

In a murin model of exposure to 4% CS f (1 h for 5 consecutive days), intraperitoneal administration of andrographolide 2 h before CSE prevents the pulmonary inflammation (BAL cell recruitment and pro-inflammatory cytokines), oxidative lung injury induces by CSE and increased the anti-oxidative response (GPX, glutathione reductase activity) in human bronchial epithelial cell by nuclear translocation of Nrf2 (Guan et al., 2013). Interestingly, the upregulation of nuclear and cytoplasmic Nrf2 was significantly increased by andrographolide after 24 h of treatment and exposure to CS while exposure to CS itself upregulated Nrf2 at 4 h but not at 24 h. Table 1 summarize the effects of Nrf2 inducers in the COPD model. Despite the promising *in vivo* and *in vitro* results, no Nrf2 inducer has thus far shown benefit in COPD patients.

### Nrf2 and Pulmonary Fibrosis

Idiopathic pulmonary fibrosis (IPF) is one of the most frequent interstitial pulmonary diseases. The incidence of IPF is 3–9/100,000 people/year in Europe and North America (Hutchinson et al., 2015). The diagnosis rests on the association of compatible histological or radiological patterns with clinical symptoms and signs including dyspnea, dry cough and velcro-like crackles on the chest examination (Raghu et al., 2018). IPF is a progressive fibrotic disease often leading to respiratory failure and death within 3–5 years (Travis et al., 2013; Raghu et al., 2018). The precise etiology of IPF remains unknown but several risk factors for IPF have been described such as aging, tobacco smoke, and environmental exposure (Lederer and Martinez, 2018). The histologic hallmarks of IPF are excessive accumulation of protein in the extracellular matrix and fibroblast foci. These pulmonary lesions are distributed within healthy pulmonary parenchyma creating spatial heterogeneity of the fibrotic lesions (Wolters et al., 2014). The pathogenesis of IFP involves multiple interactions among the lung cells, including epithelial cells, fibroblast and immune cells. Recent findings have revealed a central role for alveolar epithelial cells, especially alveolar Type II cells, in the genesis of pulmonary fibrosis in response to distal lung injury. Alteration of the alveolar epithelial barrier promotes dysregulated wound repair and harmful activation of fibroblasts (Parimon et al., 2020). This process involves several signaling pathways, including those triggered by TGF-β (Wynn, 2011). Oxidant burden and oxidative stress are additional important mechanisms for lung fibrosis (Cheresh et al., 2013). Patients with IPF exhibit increased lung oxidant burden (Rahman et al., 1999; Fois et al., 2018). The antioxidant molecule, GSH, is not increased in the lungs of IPF patients and was reportedly significantly reduced in the sputum and epithelial lining fluid of IPF patients (Meyer et al., 1994; Beeh et al., 2002). While this suggests an inadequate host response to oxidative stress, these differences may be caused by sampling issues and by the analysis of different cells.

**FIGURE 3:**
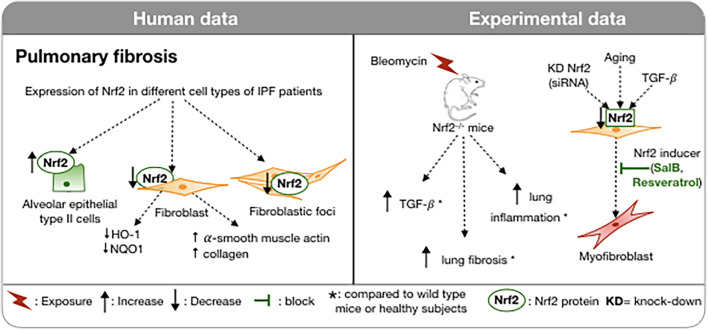
Involvement of Nrf2 in IPF in humans and in experimental models. In IPF, Nrf2 expression varies between cells: increased in type II pneumocytes and reduced in fibroblasts and fibroblast foci. In a bleomycin-induced pulmonary fibrosis model, Nrf2-deficient mice exhibit more severe pulmonary fibrosis, increased TGFβ and pulmonary inflammation compared to wild type mice. Downregulation of Nrf2 in fibroblasts induces myofibroblast differentiation. A Nrf2 inducer can inhibit this process. **↑,** Increase; **↓,** Decrease; 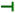, block; 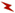, exposure; *, compared to wild type mice or healthy subjects; KD, knock-down; WT, wild type.

This redox imbalance has led to the investigation of the expression of Nrf2 in the lungs of IPF patients. Nrf2 protein was fivefold higher by Western analysis on lung homogenates of patients with IPF compared to healthy subjects (Markart et al., 2009). However, the finding was not confirmed in another study (Mazur et al., 2010). Small sample sizes in each study and the variability in the expression of Nrf2 based on the cell type could perhaps explain the discrepancies in findings since Western blot of the lung homogenate does not take account of the spatial variation of Nrf2 protein. In addition, the cellular localization of the Nrf2 protein, whether cytoplasmic or nuclear, is essential information to assess its activation. By immunohistochemistry, the expression of Nrf2 was confirmed in the alveolar epithelial type II cells and in the nuclear compartment of hyperplastic alveolar epithelium of IPF patients but was absent in the fibroblastic foci (Figure 3; Markart et al., 2009; Mazur et al., 2010). The expression of Nrf2 and its nuclear localization were reduced in fibroblasts of IPF patients; the related genes (HO-1, NQO1, epoxide hydrolase or EPHX) were reduced also. In the IPF fibroblasts, Nrf2 impaired expression was associated with an increase in expression of α-smooth muscle actin and collagen indicating a conversion to a myofibroblast phenotype. The causal link between decreased Nrf2 expression and the myofibroblast phenotype was confirmed by knock down of Nrf2 by siRNA in human fibroblasts. Interestingly, the up-regulation of Nrf2 by SFN in IPF fibroblasts and in control fibroblasts decreases the expression of collagen 1 and αSMA (Artaud-Macari et al., 2012). SFN also reduces the contraction of a collagen gel by IPF fibroblasts to a somewhat greater extent than by control fibroblasts (Artaud-Macari et al., 2012). The antifibrotic action of SFN is substantially dependent on LOC344887, a long non-coding RNA. AREs are present in the promoter and intron 1 of LOC344887. RNA-seq analysis has linked LOC344887 to fibrogenesis (Liu et al., 2021).

Stimulation of fibroblasts by TGFβ induces an increase in oxidative stress that is dependent on NADPH oxidase-4 (Hecker et al., 2009). This alteration of the redox balance is also associated with a decrease in nuclear expression of Nrf2 and differentiation into myofibroblasts (Artaud-Macari et al., 2013). Allyl isothiocyanate, also a product of cruciferous vegetables, activates Nrf2 and inhibits myofibrolast differentiation from a human fibroblast cell line (Yap et al., 2021). The differentiation of fibroblasts into myofibroblasts likely plays a pivotal role in the excessive production of extracellular matrix leading to distortion of the pulmonary architecture. These results suggest that Nrf2 could play an anti-fibrotic role by correcting this imbalance and by de-differentiating the pro-fibrotic myofibroblasts into fibroblasts. The above findings are supported by several *in vivo* studies. The most common model used to study IPF is bleomycin induced pulmonary fibrosis (Figure 3; Carrington et al., 2018). Intratracheal administration of bleomycin provokes pulmonary fibrosis through the generation of ROS including hydroxyl radicals and DNA cleaving superoxide (Claussen and Long, 1999). This model has led to the discovery of the involvement of TGF-β (Zhao et al., 2002) in the expansion of pulmonary fibrosis and the pre-clinical development of nintedanib (Wollin et al., 2015). In ICR/Sv129-Nrf2+/+ wild type mice, a single bleomycin intratracheal administration induced an inflammatory cell recruitment, in particular neutrophils and lymphocytes within 6 days, and pulmonary fibrosis as assessed by an accumulation of collagen, reflected in an elevated hydroxyproline content. Bleomycin exposure of WT mice also increased TGF-β, pulmonary expression of Nrf2 and its antioxidant response genes HO1, SOD3, and NQO1 (Cho et al., 2004). Nrf2^–/–^ mice, similarly treated, exhibit a greater pulmonary inflammation, increased expression of TGF-β, and a more substantial pulmonary fibrosis (Cho et al., 2004). Similar results have been found in bleomycin induced pulmonary fibrosis in mice lacking extracellular SOD (Fattman et al., 2003).

Nevertheless, bleomycin induced fibrosis fails to completely reproduce the pathophysiology of IPF. One important limitation is the lack of evolution of the pulmonary fibrosis. In the murine model, pulmonary fibrosis induced by bleomycin starts to resolve by 28 days after bleomycin administration (Moeller et al., 2008; Carrington et al., 2018) limiting the conclusions that can be drawn regarding pathogenesis. Other models have been used to investigate the involvement of Nrf2 in the development of pulmonary fibrosis but to date, none of the murine models reproduces in a relevant way the physiopathology of the IPF. In radiation- induced pulmonary fibrosis in the mouse, the loss of Nrf2 impairs the recovery of lung fibrosis by inhibition of the Np63 stem/progenitor cell mobilization and promotion of the radio-induced myofibroblasts derived from alveolar type 2 cells (Traver et al., 2017).

Another pro-fibrotic agent used for *vivo* experiments is paraquat (PRQ). PRQ is a powerful herbicide widely used worldwide and responsible for numerous intentional poisonings in the world (Zyoud, 2018). In humans, PRQ poisoning, regardless of the route of administration, leads to multi-organ failure, pulmonary fibrosis causing respiratory failure and death (Elenga et al., 2018). On human bronchial epithelial cells (16HBE) and in mice, it has been shown that a high dose of PRQ inhibits autophagy, cell proliferation, and promotes lung fibrosis by regulating the Keap1/P65/Nrf2 pathways (Yao et al., 2019).

Besides being involved in the regulation of the lung oxidative burden, Nrf2 also plays a role in aging (Figure 3), one of the major predisposing factors for IPF (Raghu et al., 2006) and other fibrotic diseases (Duffield et al., 2013). Increasingly evidence has linked age-related mechanisms, like genomic instability, telomere shortening, epigenetic changes, cellular loss, or senescence to IPF pathogenesis. All of these processes participate in the loss of the integrity of the alveolar epithelium and then in the aberrant activation of the respiratory alveolar epithelium that participates in the formation of fibroblastic foci and tissue remodeling (Selman and Pardo, 2014). One of the hallmarks of age-related disease is the imbalance between increases in oxidant production and/or decreases in the endogenous anti-oxidant response (Finkel and Holbrook, 2000). The ability to respond to oxidative stress through the activation of Nrf2 decreases with aging. This altered response is attributable to several age related modifications like the loss of the electrophilic response, the decrease of positive regulators of Nrf2 (PI3K, P62, CBP, BRCA1) and the increase in the negative regulators (Keap1, Bach1, and cMyc) with age (Zhang et al., 2015). Consistent with these observations, young mice (2 months) that are treated with bleomycin intra-tracheally resolve the fibrotic process more rapidly than aged mice (18 months) (Hecker et al., 2014). Furthermore, the lack of resolution is associated with an accumulation of senescent and apoptosis-resistant myofibroblasts. These specific cells were characterized by an alteration of the redox balance with increased expression of NADPH oxidase-4 (NOX4) and impaired Nrf2 antioxidant response to oxidative stress (Hecker et al., 2014). This imbalance in the NOX4/Nrf2 pathways has also been demonstrated in fibroblasts from patients with IPF (Artaud-Macari et al., 2012). Thus, Nrf2 is involved in the oxidative homeostasis of myofibroblasts whose alteration with age is associated with the persistence of fibrosis (Hecker et al., 2014).

Interestingly, daily fluctuations in endogenous Nrf2 levels seem to have an impact on the antioxidant response related to lung fibrosis. The expression of Nrf2 shows a diurnal variation in the liver and in the lungs of mice (Xu et al., 2012; Pekovic-Vaughan et al., 2014), attributable to the E-box element in the Nrf2 gene promoter. This circadian expression of Nrf2 regulates the expression of the ARE genes like *Hmox1*, *Gclm, and Gsta3*. Therefore, not surprisingly, the time of day at which exposure to bleomycin occurs impacts upon the severity of the induced pulmonary fibrosis. Bleomycin induces a more severe pulmonary fibrosis at 7 days if it is administered at the nadir of Nrf2 expression than if it is injected at the peak of circadian expression (Pekovic-Vaughan et al., 2014). The antioxidant response measured by the expression of Gclc, Gsta3, and Hmox1 also depends on the time of the day of the bleomycin injection. This opens up the possibility of a temporally targeted chemotherapeutic strategy (Pekovic-Vaughan et al., 2014).

As a plausible therapeutic target for the treatment of IPF, numerous biological compounds acting on Nrf2 have been tested for that purpose. Well-known Nrf2 activators such as SFN and resveratrol and natural bioactive components with multiple health-promoting properties including anti-inflammatory, anti-oxidative and anti-fibrotic activity have been explored. The common pharmacological properties of these substances on the lung fibrosis models are attributable to their anti-oxidant and anti-inflammatory effects, and the consequent decrease of fibrosis. Firstly, up regulation of Nrf2 by these compounds mitigates the PRQ and BLM-induced cellular oxidative stress. For instance, in BLM induced lung fibrosis in mice, the amount of ROS (Sriram et al., 2009; Liu et al., 2018), malondialdehyde (MDA) (Chitra et al., 2013; Tang et al., 2016), lipid peroxidation (Sriram et al., 2009), 3-nitrotyrosine (Yan et al., 2017), lung 4-hydroxynonenal (Yan et al., 2017) are reduced by epigallocatechin-3-gallate, salidroside, berberine, salvianolic acid B (SalB) and SFN, respectively. Similar results have been demonstrated after exposure of the transformed epithelial cell line BEAS 2B to PRQ with a decrease of the amount of ROS generated in cells treated withy resveratrol (Malhotra et al., 2010). SalB also mitigates the TGF-β induced oxidative stress assessed by the decrease of ROS and MDA levels (Liu et al., 2018). Its antioxidant activity is mediated by the PI3K/Akt/Nrf2 pathway (Tongqiang et al., 2016). All of the above compounds induced an Nrf2 up-regulation and enhanced the phase 2 enzymes response compared to the non-treated groups (Table 2). Interestingly, SalB restores the expression of Nrf2 in specific cell types that no longer express Nrf2 in IPF. In the lung biopsies of patients with IPF and as well as in BLM-induced lung fibrosis, Nrf2 is absent in the fibroblastic foci (Mazur et al., 2010; Liu et al., 2018). However, the administration of SalB in rat treated with BLM increased the expression of Nrf2 in the total lung homogenate and also in the fibroblastic foci (Liu et al., 2018).

**TABLE 2:** Effect of Nrf2 modulation in pulmonary fibrosis.

**Bioactive compound**	**Treatment administration**	**Species**	**FA (n inj; duration)**	**Outcomes**	↑ **antioxidant response**	**References**
SFN	Subcutaneous injection (0.5 mg/kg)	Mouse	BLM (1, 7, or 28 days)	- ↓ pulmonary inflammation: ↓ TNFα and IL-1β - ↓ lung induced oxidative stress (3NT; 4HNE) - ↓ lung fibrosis: ↓ fibrosis score (Szapiel Score), ↓ level of TGFβ, ↓ hydroxyproline content	NRF2^a^,^b^, HO-1^a^,^b^, NQO1^a,b^, SOD^a^,^b^, CAT^a^,^b^	Yan et al., 2017
Epigallocatechin-3-gallate	20 mg/kg body weight, once daily ip	Rat	BLM (1; 28 days)	- ↓ lung induced oxidative stress (↓ ROS and LPO) - Restores the reduced activities of the phase II enzymes NQO1 and GST induced by the BLM - ↓ pulmonary inflammation (NF-κB, IL-1β, TNFα) - ↓ pulmonary damages and ↓ the lung collagen and hydroxyproline content	NRF2^*b*^ GST^c^; NQO1^c^; SOD; CAT; GPX; GSH	Sriram et al., 2009
Salidroside	50, 100, or 200 mg/kg ip	Rat	BLM (1; 28 days)	- ↓ lung induced oxidative stress (↓ MDA) - ↓ pulmonary inflammation: ↓ inflammatory BAL cells recruitment, ↓ level of IL-6, TNFα in the BAL, inhibit IκBα phosphorylation and (NF-κB) p65 nuclear accumulation - Inhibits the BLM induced EMT assessed by ↓ *E*-cadherin and ↑ vimentin, fibronectin, and α-smooth muscle actin (α-SMA) - ↓ TGF-β1 and the phosphorylation of Smad-2/-3, a downstream target	NRF2^*b*^, HO-1^*b*^; NQO1^*b*^ SOD^c^ GSHpx^c^	Tang et al., 2016
Berberine	200 mg/kg i.p daily	Rat	BLM (1; 28d)	- ↓ lung induced oxidative stress (↓ MDA and MPO in the BAL and in the whole lung - ↓ collagen deposition assessed by hydroxyproline content, pulmonary fibrosis and ↓ level of TGF1β - ↓ pulmonary inflammation (↓ NF-κB-p65 nuclear translocation, IκB Ser32/36 phosphorylation)	NRF2^b^; SOD^c^; CAT^c^; GPX^c^; GSH^c^	Chitra et al., 2013
Salvianolic acid B	SalB at 20 mg/kg/d from 14 to 28 days	Rats and MRC-5 human embryonic lung fibroblast line	it BLM (1; 28 days)	In MRC-5 stimulated with TGF-β - ↓ markers of myofibroblast differentiation (vimentin, α SMA, fibronectin) - ↓oxidative stress (ROS and MDA) In rat exposed to BLM - ↓ markers of myofibroblast differentiation (α SMA) - Improved the induced pulmonary fibrosis	Nrf2^a,b,d^ in cells line and NRF2^*b*^	Liu et al., 2018
Quercetin	30 mM	NIH3T3; NHLFs	TGF-β	- ↓collagen production induced by TGF-β in human and murine fibroblasts - activates SMAD and MAPK pathways	Nrf2^d^, HO-1^*b*^	Nakamura et al., 2011
	Different concentrations	BEAS 2B cells;	BLM	- ↓ ROS production induced by BLM - ↓IL-8 induced by BLM	Nrf2^a^, HO-1^a^, catalase^a^, γ-GCS^a^	Veith et al., 2017
Resveratrol	10 μM	BEAS2B cells; RAW264.7; MEF	ig PRQ (10 μM)	- ↓ oxidative stress in BEAS2B cells - ↓ PRQ induced myofibroblast transformation of MEF and macrophages - ↓ PRQ induced inflammatory response (IL-6, TNFα; TNF β) in macrophages	Nrf2^*b*^; NQO1^*b*^; HO-1^*b*^ in BEAS2B treated cells	He et al., 2012
Rapamycin	0.2 mg/kg/d ig	Rats	Ig PRQ (1; 28)	- ↓ mesenchymal cell marker (Vimentin) and the EMT associated transcription factor SNAIL - ↓ mRNA expression of collagens 1 and 3	Nrf2^a,b^	Xu et al., 2017

*ip, intraperitoneally; iv, intravenously; it, intratracheal; ig, intragastric; BLM, bleomycin; PRQ, paraquat; MEF, mouse embryonic fibroblast; NIH3T3, murine embryo fibroblasts; NHLFs, normal human lung fibroblasts; MAPK, mitogen-activated protein kinase; NQO1, NAD(P)H:quinone oxidoreductase 1; γ-GCS, γ-glutamyl cysteine.*

*^*a*^mRNA expression;*

*^*b*^protein expression;*

*^c^enzymatic activity;*

*^d^nuclear localization.*

In addition to reducing the oxidative stress, the above-mentioned compounds (SFN, resveratrol, epigallocatechin-3-gallate) mitigate the pulmonary inflammation induced by fibrosing factors (Table 2). The afore-mentioned treatments increase the pulmonary expression of IL-1β, TNFα, IL-6 in BLM-induced lung fibrosis (Sriram et al., 2009; Tang et al., 2016; Yan et al., 2017) and in PRQ induced lung fibrosis in mice (He et al., 2012). EGCG, berberine, and salidroside also reduce the lung level of NF-κB, the nuclear translocation of NF-κB-p65 and the inhibition of IκBa phosphorylation, respectively (Sriram et al., 2009; Chitra et al., 2013). In RAW264.7 cells, a macrophage cell line, exposure to PRQ led to the expression of pro-inflammatory and pro-fibrotic cytokines such as IL-6 and TNFα. Treatment with resveratrol prevents the expression of IL-6 and TNFα in macrophages in a manner that is partially dependent on Nrf2. This decrease of the pulmonary inflammation is associated with a reduction of the pulmonary fibrosis severity regardless of the fibrosing factors.

The up-regulation of Nrf2 by its inducers mitigates the lung fibrosis *in vivo* assessed by the reduction of pulmonary fibrosing score (Sriram et al., 2009; Chitra et al., 2013; Yan et al., 2017), the decrease in the lung collagen deposition (Sriram et al., 2009; Nakamura et al., 2011; Tang et al., 2016; Xu et al., 2017) and the amount of lung hydroxyproline (Sriram et al., 2009; Chitra et al., 2013; Yan et al., 2017). Most of the pharmacologic agents have been tested in a preventive way but Berberin, a isoquinoline alkaloid extract, has been given before the onset of fibrosis on days 0 to 14 or after the onset of fibrosis on days 14–28 in mice administered BLM. Both drug administrations reduced the pulmonary fibrosis but the treatment was more effective when instituted before the onset of fibrosis (Chitra et al., 2013). The up-regulation of Nrf2 seems to participate in the mitigation of the lung fibrosis severity through the involvement of Nrf2 in several pro-fibrotic pathways. First, BLM exposure promotes the activation of the central mediator of fibrosis, TGF-β and of its downstream targets Smad2/3 (Della Latta et al., 2015). This activation was weakened by several Nrf2 inducers including SFN (Yan et al., 2017), salidroside (Tang et al., 2016), berberine (Chitra et al., 2013), and salvianolic acid B (SalB) (Liu et al., 2018). Secondly, Nrf2 inducers also counteract the transformation of fibroblast into pro-fibrotic myofibroblasts. Thus, SalB significantly inhibits the TGF-β1-induced myofibroblast markers like αSMA in human embryonic fibroblasts (MRC-5 cell) and decreases the αSMA pulmonary expression in rats treated with BLM (Liu et al., 2018). PRQ also promotes the trans-differentiation of WI38-VA13, a human fibroblast cell line, into myofibroblasts as assessed by an increased expression of αSMA. Resveratrol significantly mitigated this PRQ-induced phenotype in an Nrf2-dependent manner in mouse embryonic fibroblasts from WT mice or Nrf2^–/–^ mice (He et al., 2012).

Finally, Nrf2 inducers mitigate the epithelial mesenchymal transition (EMT) in lung fibrosis. An intra-gastric administration of PRQ in mice elicits pulmonary fibrosis and promotes an up-regulation of vimentin, an EMT marker as well as a down-regulation of *E*-cadherin. Treatment with rapamycin diminishes the EMT in a Nrf2 dependent manner (Xu et al., 2017). In a BLM-induced lung fibrosis model, salidroside inhibit the same EMT marker. Thus, all of these results suggest that the pharmacological activation of NRF2 by these inducers allows better control of oxidative stress, a decrease pulmonary inflammation and a mitigation of the pulmonary fibrosis by defeating several pathways. Table 2 summarize the effects of Nrf2 inducers in the lung fibrosis model.

Despite these promising results and the implication of oxidative stress in the pathophysiology of IPF, the administration of a non-specific antioxidant treatment, *N*-acetylcysteine has not shown any benefits in terms of prevention of decline in respiratory function, mortality or exacerbations in patients with moderate to severe IPF. Nevertheless, Nrf2 seems to participate in the anti-fibrotic effects of pirfenidone, one of two treatments approved for IPF in human. This molecule slows the decline in respiratory function in patients with moderate or severe IPF (Noble et al., 2011). Pirfenidone has numerous effects including an attenuation of lipid peroxidation and a clearing of the active intracellular oxygen. This suggests that pirfenidone could mitigate oxidative stress (Giri et al., 1999). Pirfenidone enhanced the mRNA and protein expressions of Nrf2, and its related genes HO-1, Gpx1 in TGF-β stimulated mouse lung fibroblasts and bleomycin-induced lung fibrosis. The upregulation of Nrf2 was associated with an inhibition of the mRNA and protein of Bach1, a Nrf2 competitive inhibitor. These results suggest that pirfenidone participates in the regulation of oxidative stress by the restoration of Nrf2/Bach1 equilibrium (Liu et al., 2017). Clinical trials of potent Nrf2 activators may be required to demonstrate therapeutic relevance of this pathway in IPF in humans.

### Nrf2 and Asthma

There is considerable biological plausibility to the hypothesis that oxidative stress is involved in asthma. Activated inflammatory cells and airway epithelial cells could both contribute to the synthesis of reactive oxygen and nitrogen species. Additionally many inhaled allergens have themselves the capacity to generate reactive oxygen species through intrinsic NADPH activity (Dharajiya et al., 2007). Allergens that provide an exogenous source of ROS are house dust mites (Ye et al., 2011) and pollens (Boldogh et al., 2005). However, not all pollens with intrinsic NADPH activity evoke allergic responses that are augmented by oxidative stress require this activity (Shalaby et al., 2013). Inhaled particulates such as diesel exhaust contain chemicals that may trigger redox cycling and are noted for their augmentation of sensitization to allergens (reviewed in Li and Nel, 2006). Oxidative stress has been associated with asthma through the demonstration of impaired antioxidant reserve in the blood of both children and adults (Rahman et al., 1996; Nadeem et al., 2003; Sackesen et al., 2008). A marker of oxidative stress, 8-isoprostane has also been shown to be elevated in the urine of severe asthmatics and to be worsened by smoking (Emma et al., 2018). Cluster analyses of an asthma cohort have revealed subsets associated with elevated 8-isoprostane in the blood (Nadif et al., 2020). Elevated hydrogen peroxide levels and 8-isoprostane can also be detected in exhaled breath condensate in asthma and are inversely correlated with lung function (Emelyanov et al., 2001; Brussino et al., 2010).

**FIGURE 4:**
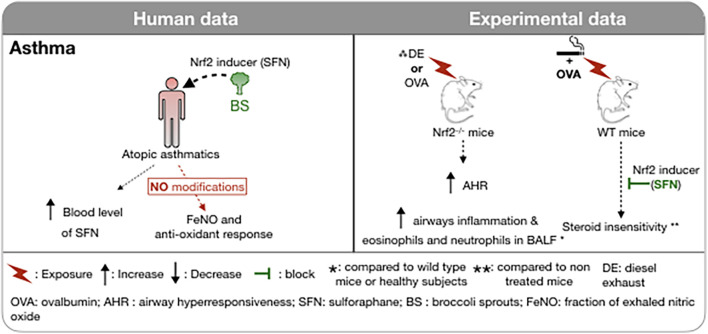
Involvement of Nrf2 in asthma in humans and in experimental models. The administration of foods rich in sulforaphane (broccoli sprouts) in atopic asthmatic subjects increased plasma SFN concentration without any modification of the FeNO or of the antioxidant response. Induction of asthma in Nrf2-deficient mice is associated with increased airway hyperresponsiveness and inflammatory cell recruitment in BAL. Administration of an Nrf2 inducer restores the corticosteroid sensitivity of ovalbumin-induced asthma concomitantly exposed to cigarette smoke. **↑,** Increase; **↓,** Decrease; 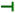, reduce; 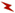, block; *, compared to wild type mice or healthy subjects; **, compared to non treated mice; DE, diesel exhaust; OVA, ovalbumin; AHR, airway hyperresponsiveness; SFN, sulforaphane; BS, broccoli sprouts; FeNO, fraction of exhaled nitric oxide; WT, wild type.

Most studies implicating Nrf2 in the protection against oxidative stress in airway disease have involved the use of epithelial cells in culture or mice exposed to allergens or irritants to model asthma driven by adaptive and innate immunity. There is convincing evidence of the role of Nrf2-dependent proteins in both allergic and irritant models of “asthma” in the mouse. Particular attention has been paid to murine models associated with neutrophilic inflammation. SFN attenuates sensitization to allergen that is promoted by diesel exhaust particles (Wan and Diaz-Sanchez, 2007) and airway responses to sensitization and challenge with ovalbumin (Park et al., 2012), house dust mite and *Alternaria* (Danyal et al., 2016; Figure 4). Steroid insensitivity caused by exposing ovalbumin sensitized and challenged mice to CS was restored by SFN, an effect that was absent in Nrf2-deficient mice (Sakurai et al., 2018).

Attempts to activate Nrf2 in the airways in humans have met with less success. Combining a potential cause of oxidative stress, namely cigarette smoking, with underlying severe asthma was not associated with an increase in expression of Nrf2-dependent antioxidant proteins in bronchial biopsies or brushings (Emma et al., 2018). A clinical trial of SFN administration (broccoli sprouts) in doses sufficient to elevate levels of SFN in the blood failed to reduce airway neutrophilia caused by ozone exposure of healthy subjects and failed to elevate phase II enzymes in the peripheral blood cells (Duran et al., 2016). Similarly, SFN administration, again using broccoli sprouts given on three consecutive days did not affect the fraction of exhaled nitric oxide or anti-oxidant enzymes in nasal epithelial cells in atopic asthmatics despite achieving substantial blood levels of SFN (Sudini et al., 2016). However, in asthmatics, daily doses of SFN significantly ameliorated airway hyperresponsiveness to methacholine challenge in 60% of the subjects (Figure 4; Brown et al., 2015). SFN was also shown to reduce the nasal allergic response and the number of inflammatory cells in nasal lavage fluid following inhalation of diesel exhaust particles, a potent source of oxidative stress, in human subjects (Heber et al., 2014).

## Conclusion

The lung parenchyma and the airways are constantly exposed to exogenous oxidants. The master transcription factor of the anti-oxidant response, Nrf2, is a crucial element for the maintenance of the redox balance. Altered Nrf2 expression has been demonstrated in many human respiratory diseases affecting the airways, in asthma and COPD, and the lung parenchyma in ARDS and pulmonary fibrosis. The loss of Nrf2 expression is associated with more severe induced respiratory diseases in animal models. Interestingly, the restoration of its expression by specific inducers in these models reduces the severity of the disease. Despite these encouraging results in cell and animal models, the administration of SFN, a potent Nrf2-activator, in patients with asthma and COPD has failed to show significant therapeutic benefit. Nevertheless, further investigation is warranted to further elucidate the role of Nrf2 in oxidative stress-driven pulmonary disease as well as the therapeutic potential of Nrf2 inducers. Perhaps, by analogy with biologics, patients with demonstrated deficient anti-oxidant responses to their disease should be selected for study in future clinical trials.

## Author Contributions

CA: conception, drafting, revising, and figure creation. TM: conception, organization, drafting, and revising. JM: organization, conception of topics, drafting, and revising. All authors contributed to the article and approved the submitted version.

## Conflict of Interest

The authors declare that the research was conducted in the absence of any commercial or financial relationships that could be construed as a potential conflict of interest.

## Publisher’s Note

All claims expressed in this article are solely those of the authors and do not necessarily represent those of their affiliated organizations, or those of the publisher, the editors and the reviewers. Any product that may be evaluated in this article, or claim that may be made by its manufacturer, is not guaranteed or endorsed by the publisher.
